# Impact of dual-tasking and balance confidence on turns and transitions: a cross-sectional study in Parkinson’s disease

**DOI:** 10.1038/s41598-026-35450-4

**Published:** 2026-01-13

**Authors:** Hanna Johansson, Niklas Löfgren, Franchino Porciuncula, Breiffni Leavy

**Affiliations:** 1 Department of Neurobiology, Care Sciences and Society, Division of Physiotherapy, Karolinska Institutet, Alfred Nobels Allé 23, 14183 Huddinge, Sweden; 2 Theme Womens Health and Allied Health Professionals, Karolinska University Hospital, Stockholm, Sweden; 3 Research and Development Unit, Stockholm Sjukhem Foundation, Stockholm, Sweden; 4 School of Health and Welfare, Dalarna University, 79 182 Falun, Sweden; 5 Department of Physical Therapy, Sargent College of Health and Rehabilitation Sciences, Boston University, Boston, MA USA

**Keywords:** Parkinson disease, Dual-task, Functional mobility, Balance confidence, Neurological disorders, Health occupations

## Abstract

**Background:**

Mobility, cognitive processing, and balance confidence impairments can negatively affect functional mobility in people with Parkinson’s disease (PD). This study aimed to examine the effects of a cognitive dual-task on functional mobility during Timed Up and Go (TUG) sub-phases involving transitions and turns. A secondary aim was to explore whether balance confidence was associated with dual-task interference (DTI) on TUG total duration and sub-phases.

**Methods:**

A cross-sectional design was employed. Participants completed TUG and TUG-COG (serial three subtractions) and inertial sensors recorded spatiotemporal data on transitions and turns. Paired samples t-tests and corresponding effect sizes (Cohen’s d) were used to compare TUG conditions. Multivariate linear regression assessed the association between balance confidence and DTI on total duration and sub-phases, controlling for gait speed and executive function.

**Results:**

People with mild-to-moderate PD (N = 94, mean age: 68.7 years) completed TUG-COG 2.7 s slower than TUG (p < 0.001, d = 0.5, DTI = 22.9%). The cognitive task led to reduced performance across TUG sub-phases, with generally stronger effects observed in turning outcomes (d = 0.25–0.45) and comparatively smaller effects observed in postural transitions **(**d = 0.02–0.38**).** Balance confidence explained variance in DTI for sit-to-stand duration (B = -−3.560, 95% CI [−5.499, −1.622], p < 0.001), whereas no effect was observed for other sub-phases.

**Conclusion:**

Dual-tasking impaired nearly all components of the TUG, prolonging total duration and altering spatiotemporal characteristics of transitions and turns. Turning was more strongly impacted by dual-tasking than postural transitions, which has relevance for fall-prevention strategies. Together, the results of this study indicate that clinicians should prioritize turning during dual-task gait training and incorporate assessment of balance confidence to better capture functional capacity in transitional movements such as sit-to-stand.

## Introduction

Functional mobility is inherent to everyday life as it involves a person’s ability to move from place to place within their environment in order to participate in daily activities^1^. In Parkinson’s disease (PD), functional mobility is negatively impacted by both motor- and non-motor symptoms^2^, and these difficulties can give rise to falls. Most falls occur in the home, and often during turning, or postural transitions such as getting in and out of a chair^3^. Free-living observations of people with PD and healthy controls have shown that each hour, on average, more than 20 turns^4^ and three sit-to-stand transitions^5^ are performed. Furthermore, real-life mobility tasks in the home often involve a dual-task, such as, talking to someone or carrying a cup of coffee, which in turn requires attention to be divided between both tasks. The gradual loss of motor automaticity in PD^6^ infers a greater challenge for this population, which adversely affects their motor performance while conducting a secondary task^7,8^. It follows that inability to perform functional mobility tasks safely, at a pace that everyday life requires, ultimately comprises participation in society. It is therefore of utmost importance that we increase our understanding of how people with PD perform common daily movements under dual-task conditions.

Functional mobility is not solely determined by motor or cognitive capacity but is also influenced by a person’s *confidence* in their ability to perform a task. Balance confidence refers to confidence in the ability to maintain balance and remain steady during activity performance^9^. Evidence suggests that balance confidence affects how people with PD negotiate transitions^10^ and turns^11^, and is also associated with increased fall frequency during these positional changes^12,13^. Understanding how and when a person’s balance confidence influences their performance during commonly performed mobility tasks during dual-task conditions can help integrate influential non-motor symptoms into clinical rehabilitation strategies.

Timed Up and Go (TUG) is recommended as the most suitable test of functional mobility for use in clinical practice and research^14^, with proven reliability in PD^15^. During TUG, a person is timed while they stand up from a chair, walk three meters, turn, walk back to the chair, and sit down. The test can also be combined with either a motor or cognitive dual-task (TUG-COG), and performance is typically characterized by total time taken to complete the tests. Resultantly, existing evidence for TUG performance focuses primarily on completion times, leaving other key aspects of movement behavior such as postural transitions and turns unaccounted for. Indeed, transitions such as sitting to walking^16^, and turning^17^ have been associated with increased risk of falling among people with PD. Assessing the extent by which increased attentional processing impacts walking transitions is critical in identifying targets for mobility training. Technical advances involving wearable inertial sensors enable the objective evaluation of how postural transitions and turns are performed during the TUG test. Sensor data may capture the specific stages of common indoor mobility tasks where a person’s movement behavior can place them at risk for falls. Preliminary evidence from PD samples suggests that, information from the different sub-phases of the TUG test may more sensitively identify fallers^18^, and predict future falls^19^, than completion times alone. Although TUG sub-phases have been studied using sensors previously, the effects of cognitive dual-tasking on subphase performance, and whether this is affected by balance confidence, is far less understood. Such knowledge could help inform clinicians when proactively targeting postural transitions and turning among at-risk groups. In this study, we used wearable sensors that quantified global and phase-specific metrics of the TUG with and without cognitive dual-tasking.

The primary aim of this study is to investigate the effects of a cognitive dual-task on functional mobility in people with PD, by comparing spatiotemporal parameters during specific sub-phases of the TUG test. We hypothesize that cognitive dual-tasking will impair TUG performance evidenced by longer durations of overall TUG and its sub-phases compared to single-task TUG. As a secondary aim, we intend to explore whether balance confidence is associated with the rate of performance decline (dual-task interference, DTI) from TUG to TUG COG, regarding total duration and test sub-phases.

## Results

### Participants

**Fig. 1:**
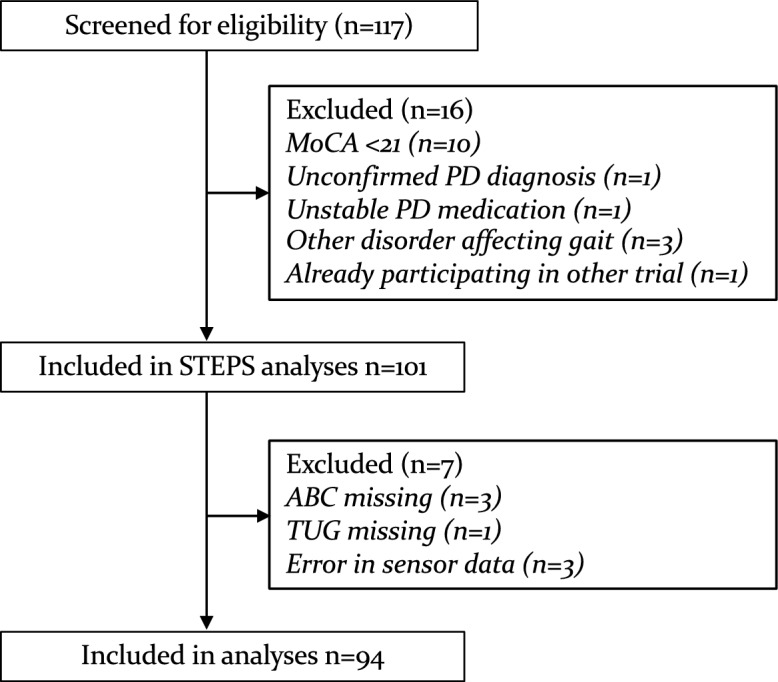
Flowchart of the inclusion process.

A total of 117 people were screened for eligibility, and reasons for exclusion from the Supported home Training in Everyday life for Parkinson’s diseaSe (STEPS) trial and from analyses for this specific report are detailed in Fig. 1. The sample of 94 included participants had an equal gender distribution, with a mean age of 68.7 years (SD 7.0) and the mean time since receiving their PD diagnosis was 6.8 years (SD 5.1). See Table 1 for demographic and clinical characteristics of the sample.

**Table 1:** Characteristics of included participants.

Characteristic, mean (SD) unless otherwise stated	N = 94
Sex, female, n (%)	47 (50.0)
Age (years)	68.7 (7.0)
Years of education	14.5 (3.0)
Years since diagnosis	6.8 (5.1)
Hoehn & Yahr stage	
1, n (%)	2 (2.1)
2, n (%)	61 (64.9)
3, n (%)	31 (33.0)
Have fallen in the last 6 months (yes), n (%)	26 (27.7)
Self-reported freezing of gait (yes), n (%)	38 (40.4)
Montreal Cognitive Assessment (0–30)	26.0 (2.4)
Trail Making Test, condition II^a^ (seconds), median (IQR)	45.9 (19.2)
Trail Making Test, condition IV^a^ (seconds)	125.7 (55.2)
Trail Making Test difference^b^ (seconds)	74.2 (47.6)
10-m walk test, usual speed (m/s)	1.3 (0.2)
Mini-BESTest (0–28)	22.8 (2.9)
Activities-specific Balance Confidence scale (0–100), median (IQR)	86.3 (22.5)
Parkinson’s Disease Questionnaire -39, single index	19.0 (10.1)
Hospital Anxiety and Depression Scale	
Anxiety (0–21)	4.7 (3.2)
Depression (0–21)	3.1 (2.4)

^a^From Delis Kaplan Executive Function System (D-KEFS).

^b^Trail Making Test condition II subtracted from Trail Making Test condition IV.

### TUG and TUG-COG performance

Based on the full sample, performance in terms of total duration, as well as eight out of ten TUG parameters of turns and transitions, significantly deteriorated with the addition of the serial subtraction task. See Table 2 for complete details on performance during TUG versus TUG-COG, as well as mean differences, effect sizes (Cohen’s d), and DTI (%).

**Table 2:** Performance on the Timed Up and Go with and without a cognitive dual-task (serial three subtractions).

	TUG (sec), mean (SD)	TUG COG (sec), mean (SD)	Mean difference (95% CI)	p-value	Cohen’s d	DTI (%), mean (SD)
Total duration (s)	9.78 (2.27)	12.46 (5.11)	2.68 (1.93–3.43)	** < 0.001**	.50	22.85 (20.92)
*Sit-to-stand*						
Duration (s)	1.05 (0.19)	1.11 (0.18)	0.07 (0.03–0.11)	** < 0.001**	.38	5.97 (14.22)
Lean angle (°)	27.27 (8.43)	27.09 (8.96)	−0.14 (−1.46–1.18)	0.834	.02	−4.57 (31.63)*
*Stand-to-sit*						
Duration (s)	0.89 (0.23)	0.91 (0.24)	0.02 (−0.05–0.09)	0.543	.09	7.48 (35.56)
Lean angle (°)	29.80 (12.71)	27.84 (11.85)	−1.96 (−3.63− −0.29)	**0.022**	.16	−7.95 (28.89)*
*Turn-to-walk (turn 1)*						
Duration (s)	2.67 (0.58)	2.93 (0.72)	0.26 (0.15–0.37)	** < 0.001**	.39	10.99 (21.04)
Turn angle (°)	187.68 (10.07)	184.58 (13.89)	−3.45 (−6.09− −0.81)	**0.011**	.28	−1.75 (6.82)
Peak angular turn velocity (°/s)	159.58 (38.68)	144.98 (43.30)	−14.72 (−19.9− −9.53)	** < 0.001**	.35	−9.21 (15.26)
*Turn-to-sit (turn 2)*						
Duration (s)	2.27 (0.59)	2.39 (0.57)	0.14 (0.02–0.26)	**0.022**	.25	10.55 (28.60)
Turn angle (°)	172.23 (14.02)	166.58 (17.72)	−6.09 (−9.05− −3.14)	** < 0.001**	.38	−3.45 (8.32)
Peak angular turn velocity (°/s)	182.32 (43.63)	161.59 (47.82)	−20.81 (−27.47− −14.15)	** < 0.001**	.45	−11.24 (16.70)

DTI = Dual task interference; TUG = Timed Up and Go; TUG COG = Timed Up and Go Cognitive.

*Median (Interquartile range).

Significant p-values in bold font.

While dual-tasking (TUG-COG), participants took 2.7 s longer (SD 3.7, p < 0.001) to complete the test compared to TUG. The sit-to-stand transition took 0.07 s longer (SD 0.18, p < 0.001), and the stand-to-sit lean angle decreased by 1.96 degrees (SD 7.18, p = 0.022). The first turn (turn-to-walk) took 0.26 s longer (0.53, p < 0.001), was 3.45 degrees wider (SD 12.8, p = 0.011) and 14.72 degrees/second slower (SD 25.04, p < 0.001). The second turn (turn-to-sit) took 0.14 s longer (SD 0.58, p = 0.022), was 6.09 degrees wider (SD 14.19, p < 0.001), and 20.81 degrees/second slower (SD 31.99, p < 0.001).

The serial subtraction task had the biggest effect on total duration (d = 0.50), followed by turn-to-sit velocity (d = 0.45), turn-to-walk duration (d = 0.039), sit-to-stand duration (d = 0.38), turn-to-sit angle (d = 0.38), turn-to-walk velocity (d = 0.35), turn-to-walk angle (d = 0.35), and turn-to-sit duration (d = 0.25). Negligible effects were noted for stand-to-sit lean angle (d = 0.16), stand-to-sit duration (d = 0.09) and sit-to-stand lean angle (d = 0.02). Sit-to-stand lean angle and stand-to-sit duration did not change during TUG-COG compared to TUG (p = 0.834 and p = 0.543 respectively).

Regarding performance on the serial subtraction task, participants had a mean (SD) response ratio (number of correct answers per second) of 1.64 (0.36) while performing this as a single-task in a seated position, and 0.45 (0.44) while performed as dual-task during TUG-COG. This corresponded to a median (IQR) DTI on response ratio of -40.1% (34.4).

### Association between dual-task interference and balance confidence

We examined the influence of balance confidence on DTI while accounting for relevant factors of gait speed and executive function. Balance confidence (ABC) showed negligible to weak correlation to DTI on the total duration and sub-phases of TUG, with Pearson r-values ranging from -0.058 to 0.294. Usual gait speed showed negligible to moderate correlations to the eight DTI, with r-values ranging from -0.032 to 0.306. Executive function (TMT difference) demonstrated moderate correlations with certain DTI variables; however, the strength of these associations varied across variables, with r-values ranging from 0.087 to 0.325. The multivariate linear models (including balance confidence, usual gait speed and executive function as independent variables) significantly explained the variance in DTI on: total duration (10.4%), sit-to-stand duration (14.1%), turn-to-walk angle (9%), and turn-to-sit angle (7.8%). The multivariate linear models with DTI on turn-to-walk duration, turn-to-walk velocity, turn-to-sit duration and turn-to-sit velocity as dependent variables were non-significant. When controlling for gait speed and executive function, balance confidence was a significant independent predictor of DTI on sit-to-stand duration only (non-standardized B -3.560, p < 0.001, 95% CI: -5.499, -1.622). For complete details of the multivariate linear regression analyses, see Table 3.

**Table 3:** Summary of multiple linear regression analysis. Independent variables included in the multivariate models were: Activities Balance Confidence scale (0–100), usual gait speed, and executive function as measured by the difference in seconds between Trail-making test A and B.

	DTI Total duration	DTI Sit-to-stand duration	DTI Tu Turn-to-walk duration	DTI Turn-to-walk angle	DTI Turn-to-walk velocity	DTI Turn-to-sit duration	DTI Turn-to-sit angle	DTI Turn-to-sit velocity
Intercept, B	43.742	36.956	18.004	− 6.670	− 25.565	− 46.007	− 7.824	− 27.506
95% CI	[4.392, 83.091]*	[8.894, 65.018]*	[− 24.034, 60.042]	[− 19.625, 6.285]	[− 55.741, 4.610]	[− 101.996, 9.981]	[− 23.825, 8.176]	[− 60.348, 5.336]
Balance confidence, B (β)	− .701 (− 0.057)	− 3.560 (− 0.438)***	− 1.691 (− 0.139)	− 0.683 (− 0.173)	0.641 (0.072)	− 0.322 (− 0.157)	− 0.774 (− 0.161)	0.358 (0.037)
95% CI	[− 3.569, 2.167]	[− 5.499, − 1.622]	[− 4.753, 1.370]	[− 1.627, 0.260]	[− 1.557, 2.838]	[− 4.399, 3.755]	[− 1.939, 0.391]	[− 2.033, 2.750]
Usual gait speed, B (β)	− 20.435 (− 0.205)	16.999 (− 0.249)*	− 3.940 (− 0.040))	6.321 (0.196)	12.693 (0.176)	36.618 (0.271)*	6.570 (0.167)	14.352 (0.182)
95% CI	[− 44.213, 3.344]	[− 33.813, − 0.184]	[− 29.302, 21.422]	[− 1.494, 14.137]	[− 5.512, 30.898]	[2.839, 70.397]	[− 3.083, 16.223]	[− 5.462, 34.165]
Executive function, B (β)	0.115 (0.260)*	0.078 (0.258)*	0.067(0.152)	− 0.007 (− 0.047)	− 0.037 (− 0.115)	0.139 (0.232)*	− 0.014 (− 0.077)	− 0.051 (− 0.147)
95% CI	[0.017, 0.213]	[0.010, 0.145]	[− 0.038, 0.172]	[− 0.039, 0.026]	[− 0.112, 0.038]	[0.000, 0.279]	[− 0.53, 0.026]	[− 0.133, 0.030]
R^2^ / Adj. R^2^	0.134/0.104	0.170/0.141	0.027/ − 0.006	0.120/0.090	0.047/0.014	0.076/0.044	0.108/0.078	0.068/0.035
F (p)	4.538 (0.005)	5.860 (0.001)	0.805 (0.494)	4.010 (0.010)	1.438 (0.237)	2.388 (0.074)	3.529 (0.018)	2.102 (0.106)
N	92	90	92	92	92	91	91	91

B = unstandardized coefficient; β = standardized coefficient; CI = confidence interval. DTI = Dual task interference.

Activities Balance Confidence scale has been reflect square root transformed which needs to be taken into consideration while interpreting these results. *p ≤ .05, **p < .01, , ***p < .001.

## Discussion

The primary aim of this study was to investigate the effects of a cognitive dual-task on functional mobility by comparing spatiotemporal performance parameters during specific sub-phases of the TUG test. As hypothesized, TUG performance deteriorated while dual-tasking, evident in total duration and most parameters related to postural transitions and turns. As a secondary aim, we investigated whether balance confidence was associated with dual-task interference on the total duration and sub-phases of TUG. Findings indicate that balance confidence significantly accounted for variability in dual-task interference, particularly during the sit-to-stand phase of the TUG, while its influence was not evident in other sub-phases.

In line with our hypothesis, performance declined while dual-tasking regarding both total completion time and most elements of postural transitions and turns. The serial subtraction task exerted most pronounced influence on overall performance, yielding a medium effect size for total duration (d = 0.50), which exceeded the effects observed for individual sub-phases. Although both TUG and TUG-COG have been extensively explored in the past, studies where the differences between the two tests have been significance-tested are surprisingly rare^20,21^. Instead, previous studies in people with PD have used the total duration of each test to compare between groups, such as between PD/healthy controls^22^, motor symptom phenotypes^23,24^, freezers/non-freezers^25^, groups with/without mild cognitive impairment^26^ or fallers/non-fallers^27^, but not between TUG and TUG-COG duration per se. While comparing groups based on total test duration is informative, investigation of between-condition effect can also provide clinically relevant insights, e.g., regarding automaticity and task prioritization. In this context, the finding that dual-task effects were most pronounced in overall test performance rather than in individual sub-phases is noteworthy. Although total test duration showed a larger dual-task effect than the sub-phases, it can be argued that this measure reflects the cumulative impact of impairment across all components. Clinicians commonly rely on total TUG time as a practical measure, and assessing overall performance does not require costly sensor equipment. This indicates that meaningful evaluation of dual-task effects can be achieved using standard clinical procedures, supporting the continued use of this approach in routine practice. However, improving general mobility, as indicated by improved TUG or TUG COG performance, likely requires targeted training not only on walking, but also on turns and transitions to achieve meaningful gains in overall mobility.

Findings revealed that the effect of the serial subtraction task was also evident in the sub-phases of TUG-COG compared to TUG. The sub-phases sit-to-stand and both turns seemed to have comparable magnitudes based on effect sizes of duration (d = 0.25–0.39). However, angular outcomes had higher effect sizes during both turning phases (d = 0.28–0.45) than during sit-to-stand (d = 0.02) (d ranged between 0.02 and 0.38 for postural transitions, and between 0.28 and 0.45 for turns). Overall, the findings indicate that transitions were less susceptible than turns to the effect of the added cognitive dual-task, possibly due to a shift toward periodic execution of the movement components within transitions instead of performing them simultaneously. Indeed, people with PD tend to sequence these movements and come to a full standing position before initiating gait, while healthy controls initiate gait closer to seat-off^28^. Compared to healthy controls, people with PD have also been shown to use an exaggerated hip flexion strategy while performing sit-to-stand, possibly in order to minimize demands on balance^29^. It is possible that by dividing postural transitions into different motor sequences and using a pronounced hip flexion strategy, the participants’ balance became less challenged, making them less vulnerable to the added cognitive load. Overall, stand-to-sit was the subphase least affected by the serial subtraction task (negligible effect, d = 0.09 and 0.16). Although somewhat speculative, this may be due to an early termination of counting once participants had approached the chair and turned around, thereby performing the stand-to-sit transition without continuing to dual-task. However, without video recording, verification of this theory is not possible. Another plausible explanation, previously observed in older adults, is that adding a dual-task to TUG may lead to a more abrupt stand-to-sit transition^30^. The tendency for quicker, less controlled stand-to-sit movements under dual-task conditions may explain why transition times remained unchanged while lean angle varied, as observed in the current study.

Turning behavior during TUG is an important outcome in PD, with the potential to predict future falls^17^, and discriminate between different levels of disease severity^31^ and motor symptom profile^32^. Compared to healthy controls, individuals with PD tend to execute turns more slowly and with increased width, reflecting a strategy that prioritizes postural stability over speed or directional accuracy^33^. In the present study, adding a serial subtraction task further amplified this pattern, with participants performing turns that were both wider and slower than during single-task conditions, indicating a compensatory strategy during dual-task demands. Previous research further indicates that individuals with PD typically maintain a spin-turn strategy rather than switching to a more stable step-turn when dual-tasking^34^. It could be argued that if people with PD turn in an unsafe manner, particularly under dual-task conditions, it follows that balance confidence could play an influential role in turn execution. Interestingly, this study did not indicate that balance confidence was associated with the rate by which turn performance declined when performing a dual-task. However, although these findings suggest that balance confidence may not influence the DTI on turning in this sample, they underscore the importance of investigating turning behavior during dual-task conditions to better understand fall risk and inform targeted prevention strategies.

While no effect was observed in other sub-phases, balance confidence significantly explained variation in dual-task interference during the sit-to-stand phase. This finding introduces a psychological dimension to previously reported biomechanical and clinical factors, such as weight-shifting difficulties, bradykinesia, and balance deficits, that are central to the progressive deterioration of sit-to-stand performance in people with PD^35^. Given how frequently sit-to-stand movements occur in daily life^5^ and the common presence of dual-tasking during these activities, it is crucial to understand the full scope of underlying difficulties, and to identify strategies for targeted training interventions. Future research should explore these mechanisms in greater detail and evaluate the effectiveness of interventions that address both motor and cognitive demands during sit-to-stand tasks.

Some limitations should be addressed. Our study primarily focused on postural transitions and turns, which are related to mobility impairments and falls in people with PD. However, data on other sub-phases of TUG (e.g. gait initiation, straight-walk) were not collected, thus the entirety of the TUG is not characterized in this study. In addition, although we collected data for potentially important turning outcomes, we lack information on turn strategies used, and do not report number of steps or other metrics that could add to the interpretation of whether a turn was “safe” or not. Together, this data would have provided important and completive clues to our results. It is also important to note that the sequence in which the tests were administered may have introduced a systematic bias which affected the outcomes. By recording the TUG in the single-task condition first, two opposing effects could have occurred: (1) dual-task interference may have been overestimated if participants experienced fatigue after completing the initial test, or (2) dual-task interference may have been underestimated if the initial single-task trial provided additional practice, enabling participants to perform the motor component of the TUG more efficiently during the dual-task condition. Further, participants in the current study had a median ABC score of 86.3, indicating high overall balance confidence, which should be considered when interpreting the findings. Future studies should explore whether other patterns of association between balance confidence and dual-task interference on functional mobility emerge in populations samples with lower balance confidence. Despite these limitations, this study, which included a relatively large sample of individuals with PD, was among the first to examine the impact of a cognitive dual-task on TUG sub-phases and to comprehensively assess the role of balance confidence.

In conclusion, the results demonstrate that dual-tasking adversely affects nearly all components of the TUG, prolonging overall duration and altering spatiotemporal characteristics of both transitions and turns. Turning was disproportionately impacted compared to postural transitions, underscoring its relevance for fall-prevention strategies. Collectively, these findings highlight the need for clinicians to incorporate turning tasks into dual-task gait training and to assess balance confidence as part of routine evaluation to ensure a more comprehensive understanding of functional capacity during transitional movements such as sit-to-stand.

## Methods

### Study design and setting

This cross-sectional study used baseline data from the STEPS trial, registered at clinical trials (NCT05510739, first posted August 22, 2022). A study protocol for the STEPS trial has been published previously^36^. Data for the current study was collected in a primary care setting (Stockholm Sjukhem) in Stockholm, Sweden, from September 2022 until October 2023.

### Ethics declaration

All participants received verbal and written information before providing informed, written consent to participate, and the study was approved by the Swedish Ethical Review Authority (Dnr:2022-02, 979-01). All methods were performed in accordance with the relevant guidelines and regulations, and in accordance with the Declaration of Helsinki.

### Participants

Participants were recruited via advertisement on social media platforms, and by advertisement in patient organization publications. Eligibility criteria for the STEPS trial included a diagnosis of idiopathic PD for ≥6 months, stable PD medication ≥ 3 months, age ≥50 years, ability to walk indoors continuously ≥ 6 minutes without a walking aid, ≤2 falls in the last month, and a score of ≥21 on the Montreal Cognitive Assessment (MoCA)^37^. Participants were excluded if they had any other neurological or orthopedic disorders that impeded gait or balance. The assessments were carried out during ON stage of levodopa medication, and the order of the tests were the same for all participants.

### Procedures and outcomes

#### Primary outcome

Functional mobility under single and dual-task conditions was assessed by TUG and TUG-COG, respectively. At a given start signal, participants were to stand up from a chair (*sit-to-stand*), walk three meters, turn (*turn-to-walk*), walk back to the chair, turn (*turn-to-sit*), and sit down (*stand-to-sit*). Each test was conducted once at “comfortable” pace and prior to assessment participants had one practice trial (not included in analysis). During TUG-COG, a serial three subtraction task (counting backwards by threes from a start number between 90 and 100) was added as the cognitive dual-task. During testing, the order was the same for all participants: 1) practice trial of the TUG; 2) TUG; 3) practice trial of serial subtractions; and 4) TUG-COG.

**Fig. 2:**
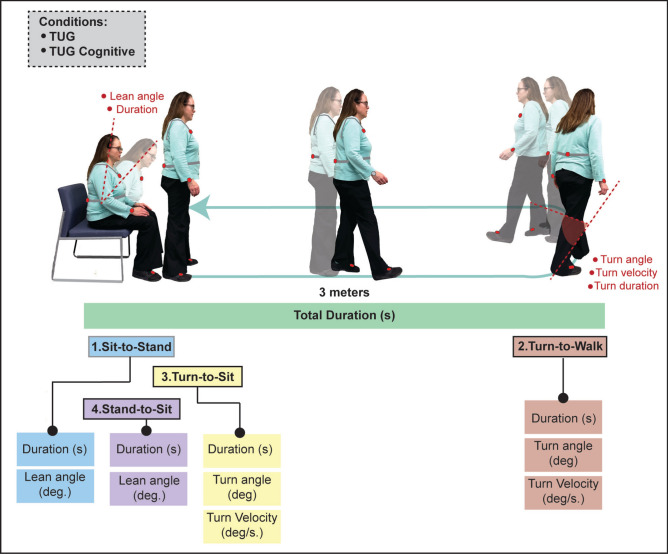
Illustration of spatiotemporal mobility parameters analyzed during sub-phases of the Timed Up and Go. Sensor placement is depicted in red.

The APDM Mobility Lab inertial sensor system (Opal, APDM Inc.) was used during tests of gait and functional mobility to capture spatiotemporal movement parameters. A total of six sensors were placed on wrists, feet, lumbar and sternum. The outcomes used for the current analysis related to performance of the total test (duration, seconds), as well as postural transitions (sit-to-stand and stand-to-sit) and turns (turn-to-walk and turn-to-sit). Duration (seconds) and lean angle (angular range of motion of the trunk, degrees) was captured during postural transitions, while duration, turn angle (rotational angle of the turn, degrees) and peak angular velocity (degrees/second) was captured during turns. See Fig. 2 for an overview of the sub-phases of TUG/TUG-COG, and each respective spatiotemporal parameter captured with the inertial sensors.

#### Clinical characteristics

Demographic information regarding sex, age, years since PD diagnosis, years of education, and fall-history over the last 6 months, was collected. Motor symptom progression and level of disability was determined by the Hoehn and Yahr (H&Y) scale^38^. Gait was assessed with the 10 m walk test, a test with excellent reliability (ICC = 0.92)^39^. The Mini Balance Evaluation Systems Test (Mini-BESTest)^40^ which has good person and item reliability (ICC = 0.90 and 0.99 respectively) and internal construct validity in people with PD,^41^ was used to assess balance performance. Self-reported balance confidence was collected with the Activities-specific Balance Confidence Scale (ABC scale, ranging from 0 to 100 with higher scores indicating higher balance confidence)^42^. Self-reported symptoms of freezing of gait (FOG) in the last month were collected using item 1, appendix 1, of the New Freezing of Gait Questionnaire (NFOG)^43^. Well-being and health status was assessed by the Parkinson’s Disease Questionnaire–39 (PDQ-39)^44^ and Hospital Anxiety and Depression Scale^45^. Trail Making Test (TMT), second and fourth condition, from Delis Kaplan Executive Function System (D-KEFS) were collected, and the difference in seconds between the two conditions were used as a measure executive function.

### Data analysis

A power calculation was conducted for the larger scale RCT from which this study used baseline data,^36^. Normality of variables was assessed by skewness and kurtosis values, Kolmogorov–Smirnov values, and by visual inspection of QQ-plots and histograms. Mobility Lab software (Version 2) was used to analyze spatiotemporal movement data. Spatiotemporal gait data was processed using Mobility Lab software (Version 2) which has been validated for use in PD^46^. Data for turns and transfers were extracted from individual Mobility Lab CSV files, using parameters based on established APDM algorithms. Statistical analyses were conducted using IBM SPSS Statistics for Mac version 28 (IBM Corp).

Performance over TUG and TUG-COG was examined using descriptive statistics, whereas comparisons between performance in the two conditions were explored using paired samples t-test. Effect sizes between the paired samples were expressed in Cohen’s *d*, and calculated by the formula $d=tc\surd (\frac{2\left(1-r\right)}{n})$ , where tc is the *t*-test statistic for mean differences for pairs of observations with α = 0.05 and degrees of freedom is 2n – 2, *r* is the Pearson correlation coefficient across pairs of observations, and *n* is the number of observations, as suggested by Dunlap et al.^47^. When interpreting the Cohen’s *d* effect sizes, the following cutoffs were used: small effect (*d* = 0.2), medium effect (*d* = 0.5), and large effect (*d* = 0.8)^48^. The ratio by which performance changed from TUG to TUG COG, i.e., the DTI, was calculated as proposed by Kelly et al.^49^.

$$DTI \left( \% \right) = \left( {\frac{TUG COG - TUG}{{TUG}}} \right) \times 100$$

For the exploratory secondary aim focusing on the impact of balance confidence, only the eight TUG subcomponents that demonstrated an effect size ≥ 0.2 in the paired samples t-test were retained, reducing the original set of 11 comparisons. The distribution of ABC scores in the current sample was negatively skewed (with most scores clustered near the upper end of the scale); therefore, a reflect and square root transformation was applied to enable the use of parametric statistics. Bivariate correlations and multivariate linear regression analyses were used to explore whether balance confidence (i.e., ABC) was associated with DTI on the eight different TUG subcomponents. Potential confounding variables included age, disease duration, usual gait speed and executive function (TMT difference). Age and disease duration were removed from further analysis since they did not show a correlation to any of the DTI variables and the final models were therefore adjusted for usual gait speed and executive function.

## Data Availability

The dataset contains personally identifiable information and is therefore subject to ethical and legal restrictions on public sharing, according to Swedish laws. However, questions concerning datasets and analysis can be answered by the corresponding author.
